# Correction: An exploratory study of behavioral traits and the establishment of social relationships in female laboratory rats

**DOI:** 10.1371/journal.pone.0352127

**Published:** 2026-06-22

**Authors:** Shiomi Hakataya, Noriko Katsu, Kazuo Okanoya, Genta Toya

In S1 Fig, the reported Eigenvalues were incorrect. In S3 Table, the 95% confidence intervals of correlation coefficients of PC3 were also incorrect. Please view the correct S1 Fig and S3 Table below.

In the Personality structure subsection of the Results, there is an error in the second sentence of the first paragraph. The correct sentence is: The Kaiser criterion supported the adoption of the first four components (S1 Fig).

## Supporting information

S1 Fig Scree plot derived from the PCA for the items of five behavioral tests. (TIF)

S3 Table The 95% confidence intervals of correlation coefficients between principal component values and the indices of social interactions standardized by group average value. Inverted positive/negative values were shown for PC3 for ease of interpretation.
(TIF)
