# Supplementary figures and images for: An exploratory study of behavioral traits and the establishment of social relationships in female laboratory rats
Source: PLoS One. 2023 Dec 4;18(12):e0295280. doi: 10.1371/journal.pone.0295280 (PMC10695365; doi:10.1371/journal.pone.0295280)

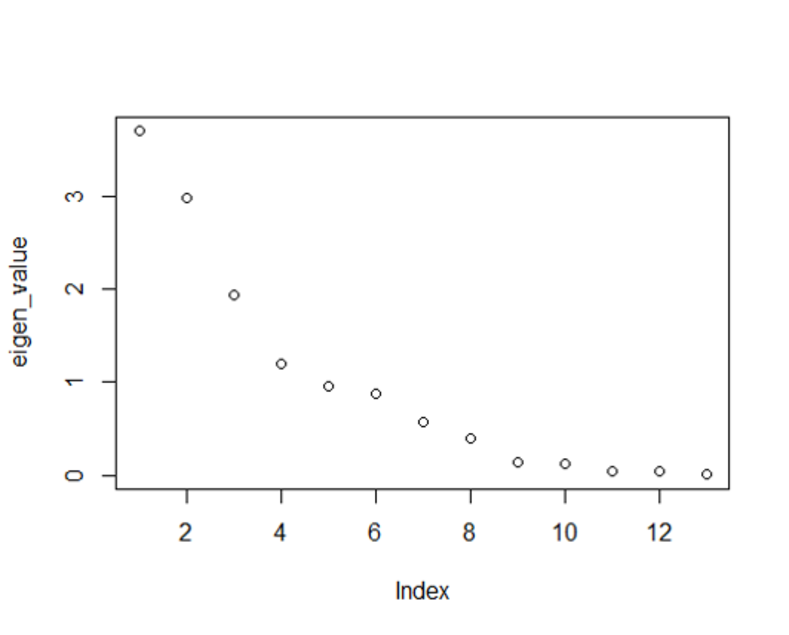

Supplement: S1 Fig — (TIF) [file pone.0295280.s001.tif]

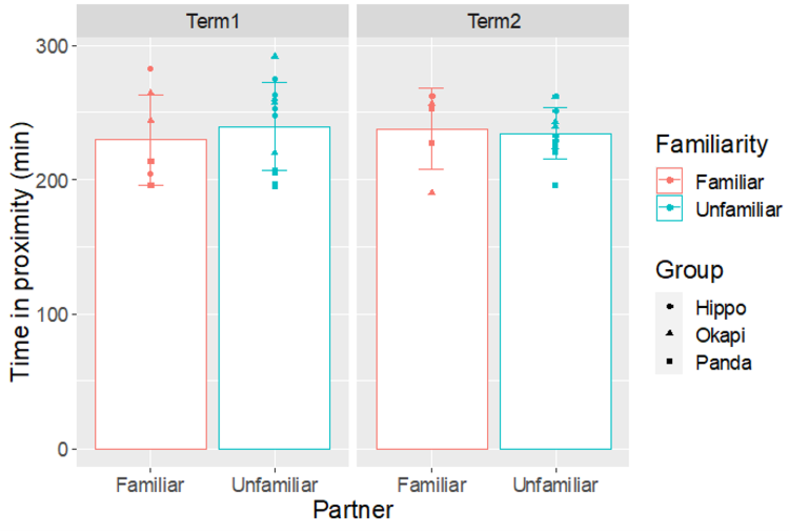

Supplement: S2 Fig — There were no significant effects of familiarity (N = 12, 18 dyad, LMM and likelihood ratio test, term1: χ2 (1) = 2.188, p = 0.139, term2: χ2 (1) = 0.333, p = 0.564). (TIF) [file pone.0295280.s002.tif]

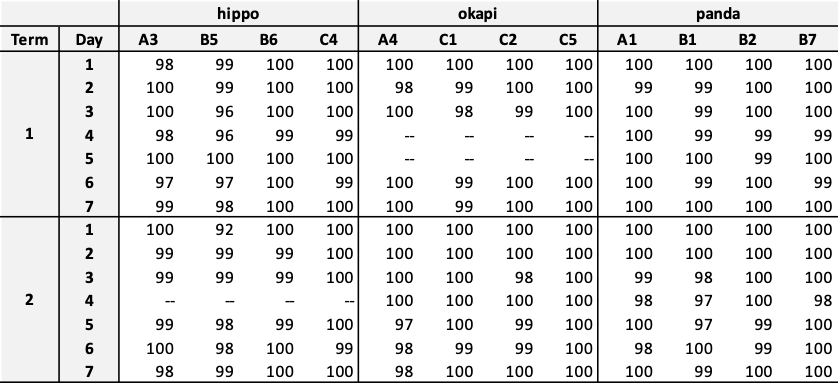

Supplement: S1 Table — (TIF) [file pone.0295280.s003.tif]

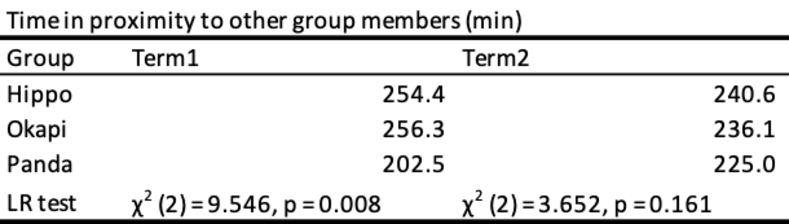

Supplement: S2 Table — Significant differences by LMMs and likelihood ratio test was found in term 1 (N = 12). (TIF) [file pone.0295280.s004.tif]

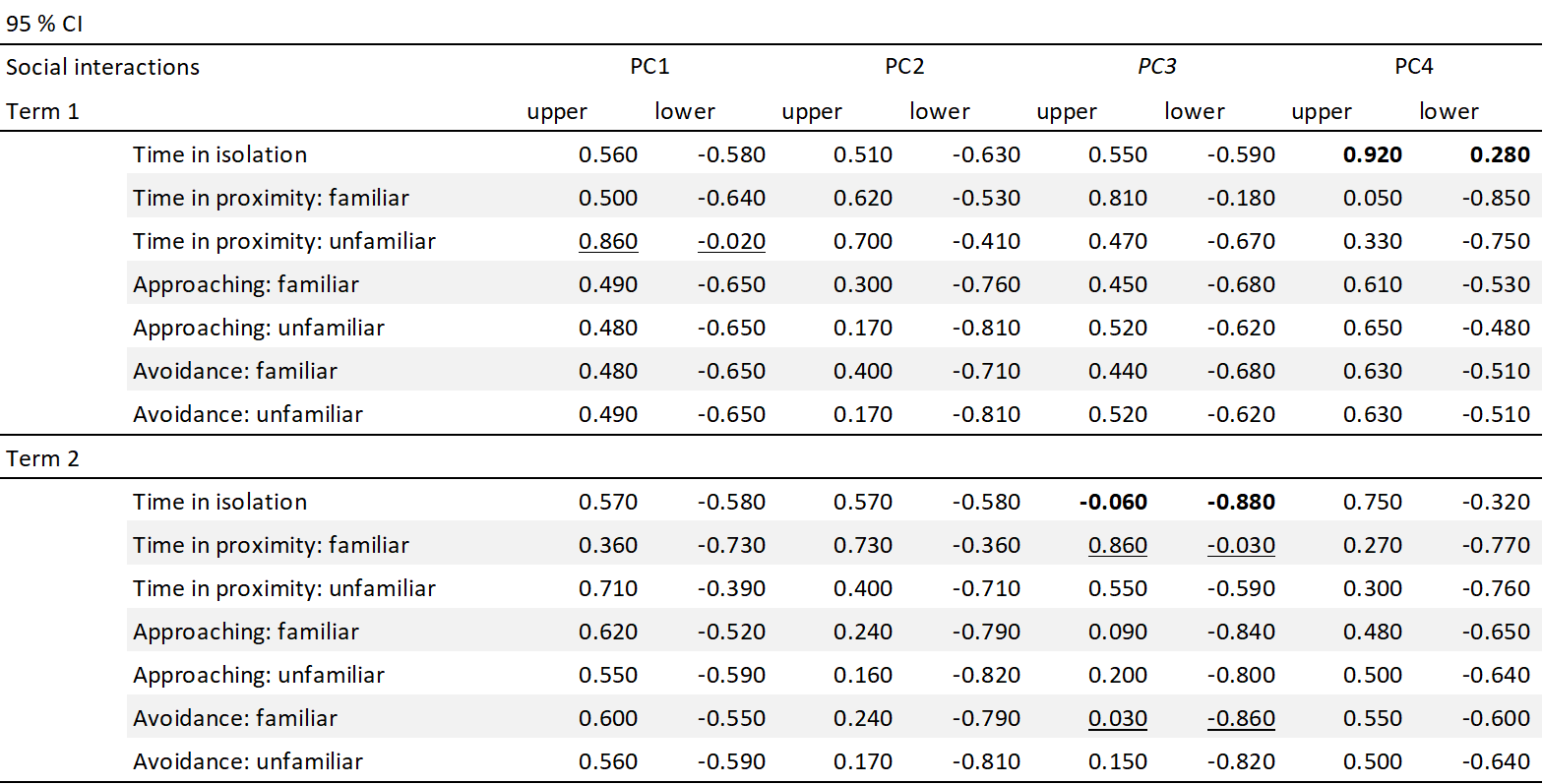

Supplement: S3 Table — Inverted positive/negative values were shown for PC3 for ease of interpretation. (TIF) [file pone.0295280.s005.tif]
