# Supplementary material for: An exploratory study of behavioral traits and the establishment of social relationships in female laboratory rats
Source: PLoS One. 2023 Dec 4;18(12):e0295280. doi: 10.1371/journal.pone.0295280 (PMC10695365; doi:10.1371/journal.pone.0295280)
Supplement: S1 File — (DOCX) [file pone.0295280.s006.docx]

**Materials and methods**

**Behavioral tests**

**Glove test**

Animals were placed into the test cage and left there undisturbed for a five-minute habituation period. The glove test consisted of six steps, each lasting twenty seconds:

1. The experimenter opened the door and placed her hand inside the cage, close to the opening.
2. The experimenter moved her hand to the middle of the cage and put it down on the cage floor.
3. The experimenter moved her hand the back of the cage and put it down on the cage floor.
4. The experimenter attempted to gently stroke the animal with her right palm.
5. The experimenter attempted to pick up the animal and hold it in her hand.
6. The experimenter put the animal down and withdrew her hand from the cage.

Recorded videos of the glove test were scored using tanaMove_v0.09 (event recording software by keyboard typing). The scorer pressed the assigned key upon observing a given behavior. The minimal key input duration was 0.05s. The total duration of approach, avoidance, and handling were measured. No aggressive behaviors were observed during the test. The absolute durations were used in the principal component analysis. Definitions of the behavioral indices are as below.

・Approach: if the rat stretched or moved toward the hand, sniffed the hand, or touched the hand without being handled or touched by the experimenter..

・Avoidance: if the rat moved away from the hand without being touched or handled by the experimenter.

・Handling: If the rat remained in the hand without aversion after the experimenter picked up the animal.

**Novel object test**

Before the novel object test, animals were habituated to the arena for two fifteen-minute periods over two days.

After the test trial, cage-mates of the tested animal were transferred to the small cage for the novel object test, and then the tested animal was returned to the home cage. This procedure was chosen to prevent the tested animals from contacting their cage-mates, because social contact may influence the cage-mates’ consumption of the novel food.

**Three-chamber test**

Before the test, subjects were habituated to the testing arena for two ten-minute periods over two days. Stimulus animals were also habituated to the restraint chambers in four 30-min habituation trials (during the last habituation trial, the restraint chambers were placed into the arena).

**Behavioral tracking in a group**

**Recording Data Processing**

Due to GPU processing performance limitations during video analysis with DeepLabCut, the video shot at 3840x2160 with 30 fps was downscaled to 1920x1080 with 15 fps using ffmpeg.

**DeepLabCut (DLC)**

Eight body points (nose, head, right ear, left ear, neck, upper body, lower body, and tail base) were tracked using DeepLabCut 2.2. A deep neural network model for tracking was created for each rat using 140 recorded images randomly selected from the first day of term 1 of group housing; although DLC is capable of tracking multiple individuals, this function was not used in this study. Only the upper body was used to estimate the location of individuals, as it is less likely to be obscured by other individuals and is thus more likely to be tracked accurately. Since annotating multiple points improves tracking accuracy, 8 body locations were tracked per individual. The construction procedure for the model is as follows:

1. Create a project for each individual on the DLC.
2. Train the model 500,000 iterations using 50 recorded images of the first day of the group housing period.
3. Use the first training model to track each individual on the first day and estimate location.
4. Extract 30 frames with low estimation likelihood, correct the position, and merge with iteration0 training data.
5. Train the model 500,000 iterations using the updated image data.
6. Steps 3 to 5 are repeated until the increase in likelihood reaches a plateau (usually 3 repetitions).

**Analysis of DLC coordinates**

X and Y coordinates estimated from DLC were modified to correctly track the individual positions by a correction program written in C++ (see OSF for the code). The process for correction consists of a combination of the following parts and iterations. See the program directly for the specific implementation and parameter settings of each process.

- Jump detection: The average velocity (v_ave) of a fixed interval is calculated in time window t_w. If the distance traveled (i.e., velocity in one step) in the frame immediately following the interval exceeds the coefficient vc_lim x v_ave, the frame is considered a jump and the likelihood is reduced. Only coordinate data with a likelihood greater than 0.8 are used to calculate the average velocity. Frames with speeds greater than v_unreal, which are not realistically possible, are also excluded.
- Jump correction: Correction based on estimated likelihood and movement speed. Frames with a likelihood greater than 0.8 are used to correct frames with a likelihood of less than 0.8. There are two types of corrections:
  - Linear interpolation using frames immediately before the frame to be corrected and frames at a future time t_f.
  - Velocity interpolation using the average velocity of the interval that follows the frame to be corrected.
- Outlier parts detection: Positions such as the tip of the nose and tail base are more likely to miss location estimates. Outliers are detected by using data from parts with higher estimation likelihood. Within a frame, if body parts are longer than a certain distance from other parts, the likelihood estimation of those parts is reduced.
- Outlier parts correction: For a part with a low likelihood of position estimation in a frame, the distance from the other parts in the frames before and after it is calculated. If the distance is less than the length of the rat's body (300 mm) and is not the same position as the other parts, a score is given. The scoring is conducted in the following two methods.
  - A) Calculate the distance of the part from each of the other body parts in the frame at t-1 immediately before the frame t to be corrected and from each of the other body parts in the frame at a future time t+x, and award points to any that are not outliers.
  - B) Calculate the distance of the part from each of the other body parts in the frame at t+1 immediately after the frame t to be corrected and from each of the other body parts in the frame at a past time t-x, and award points to any that are not outliers.
  - If the score for either method is zero, redo after x +1.

The scores calculated by the above two methods are compared, and the method that produce the higher score is adopted for outlier parts correction. If the method A is adopted, linear interpolation is performed using the two frames t-1 and t+x. If the method B is adopted, the two frames t+1 and t-x are used for linear interpolation.

- Original data completion: As the correction is applied repeatedly, data that was highly accurate at the time of the DLC original data becomes misaligned. To correct this, if the coordinates of the corrected data and the original data are close, the data is restored to the coordinates of the DLC original data.
- Rolling compensation: Frames with low likelihood that could not be corrected by any of the above correction methods are filled with values from immediately preceding frames with a likelihood greater than 0.8.

To evaluate the accuracy of each coordinate, we extracted 100 frames every 324 seconds from 9 hours of video tracking and scored the estimation performance of individual location based on whether upper body, which generally tends to be more accurate, fell within the contour of each individual. The test results showed that on almost all dates, the estimation accuracy of each individual’s upper body was 96% or higher (S1 Table).

Based on video resolution and cage size, the actual distance between coordinates was calculated (0.72 mm/px). The moving velocity of individuals was calculated based on the estimated coordinates of a point on the upper body, and activity information and inter-individual relationships were quantified. Estimates with a likelihood 0.8 or higher were adopted as credible data. The moving velocity was calculated as a scalar value averaged over a time window of 15 frames before and after a certain frame. Based on this moving velocity, the total active time, total distance traveled, and average moving velocity during activity were calculated for each individual, for each day. The total active time counted frames with a moving velocity between 75 mm/s and 3000 mm/s, taking into account dispersion of estimated location information and jumps due to misestimation. The total distance traveled and average moving velocity were calculated from the information in these frames.

As for inter-individual relationships, we calculated the time in proximity, time in isolation, and the number of times that individuals approached and avoided one another. The threshold for proximity determination was set at a distance of 200 mm or less between the upper body of each individual. Time in isolation was calculated by defining it as the time that any individual was separated from all the other individuals by more than 400 mm. If the rat got closer than 150 mm to another individual, it was considered to have approached that individual. As a buffer to prevent false counts, the next approach was not counted until the animal was farther apart than 250 mm from the target. If the rat moved farther than 250mm from another individual, it was considered to have avoided that individual. The next avoidance was not counted until the animal was closer than 150 mm to the target. The threshold for the inter-individual relationship index was set based on the head and body length of the subjects (approximately 200mm): 200mm for proximity, 400mm for isolation, 150mm for approach, and 250mm for avoidance.
