# Supplementary material for: An exploratory study of behavioral traits and the establishment of social relationships in female laboratory rats
Source: PLoS One. 2023 Dec 4;18(12):e0295280. doi: 10.1371/journal.pone.0295280 (PMC10695365; doi:10.1371/journal.pone.0295280)
Supplement: Attachment — Submitted filename: Review PONE-D-23-27375.pdf [file pone.0295280.s007.pdf]

As written before, below are my comments organized by page and line.

Page 4, line 48: The authors should specify which kind of social bonds are reported here. For example, parenting will have a different effect on the longevity of female baboons than bonds based on conflicts.

Page 5, line 59: The approach of the authors to personality is rather too simple and distant from current theories. Personality is now understood as more than just a set of timely-conserved behaviors (see Lages and McNaughton, 2022; Revelle, 2007) as bottom-up and top-down approaches are often combined in attempts to characterize personality in non-human animals and in translational research (for a background: Big-5, HiTOP, and Eysenck/Gray/RST theories). The authors should care for a more detailed approach of Personality and then define the scope in which they will work with.

Page 5, line 59: While these axes are certainly used to characterize personality traits in non-human animals, they are not the only ones. For example, while in fishes these axes can characterize well populations, they may not be suitable in describing individuals (see Carter et al., 2013; Sánchez-Tójar et al., 2022). On the other hand, these axes may be too simplistic to describe the personality of rodents (see Michelini, G, 2021). The authors should cite other theories of organization and then concentrate on their chosen approach while acknowledging its limitations.

Page 5, line 63: Although a very brief explanation of each axis is given in parenthesis, in the following subsections they must be defined and characterized more thoroughly.

Page 5, line 71: As stated before, the text can benefit from a more descriptive explanation of the meaning of each term (in this case, exploration and activity).

Page 6, page 82: it lacks the species nomenclature of “Eastern garter snakes”.

Page 6, line 82: As the authors did in the topics before, here the text could benefit from citation of results in different species. Also, citations of papers comparing natural observations x experimental approaches, as this was a topic approached earlier by the authors.

Page 8, line 125: Do differences in tameness in lab animals reflect differences between wild and (general populations of) lab animals? If the authors chose to use this measure, this correlation should be showed before.

Page 9, line 133: While the choosing of these tests is adequate in my point of view, the authors should acknowledge the dependence between the difference measures. For example, the open field test was used for measure activity, however these results can also be impacted by boldness. None of the tests is "clean" enough to be a reflection of just one personality trait. It can be stated in the introduction and then discussed taking into account the results obtained with the principal components analysis (as the authors have already started doing).

Page 10, line 151: Why only females were used? The authors should justify it in the introduction (or alternatively in methods).

Page 10, line 166: Typo – “names” > “named”.

Page 13, line 203: Please specify how many hours of food deprivation.

Page 14, line 224: “first” can be changed to “at first”.

Page 14, line 225: “second” can be changed to “later” or “next”.

Page 20, line 336: To which tests were the DLC analysis applied to? All? The authors should specify.

Page 30, line 466: Although not reaching the cutoff criteria, comments can be made about the association of PC2 with proximity to unfamiliar groups, as it is related to exploration and boldness itself (as the authors correctly pointed out in the discussion).

Page 33, line 521: Which other inferences can one have with the three-chamber test? Do the authors believe this test should not be used to test sociability in general or is this a particularity of their experimental setup? The authors should make a comment about it, especially because this test has been classically used for measuring sociability (see Rein B, 2020; Kaidanovich-Beilin, 2011)

Page 36, line 563: Not only here, but throughout the discussion the authors must comment on their choice for using solely female rats and how their results could be extrapolated (or not) to both sexes.

Page 37, line 583: As reasonable as this hypothesis seems, the text could be improved with citations that show the decrease of the influence of the exploration-avoidance and shyness-boldness reduced over time.
