# Supplementary figures and images for: Correction: An exploratory study of behavioral traits and the establishment of social relationships in female laboratory rats
Source: PLoS One. 2026 Jun 22;21(6):e0352127. doi: 10.1371/journal.pone.0352127 (PMC13286133; doi:10.1371/journal.pone.0352127)

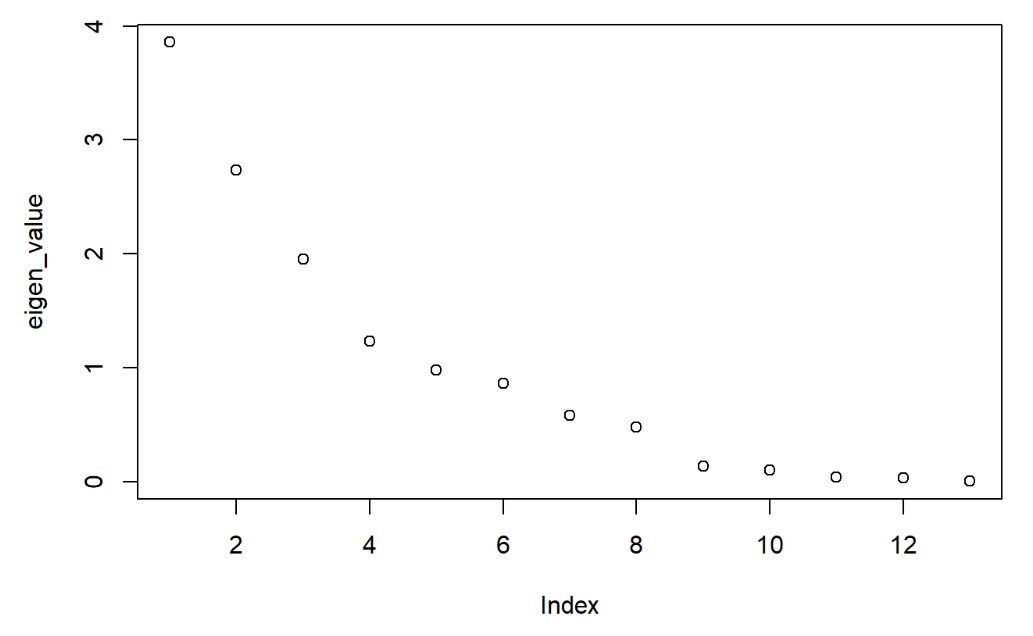

Supplement: S1 Fig — (TIF) [file pone.0352127.s001.tif]

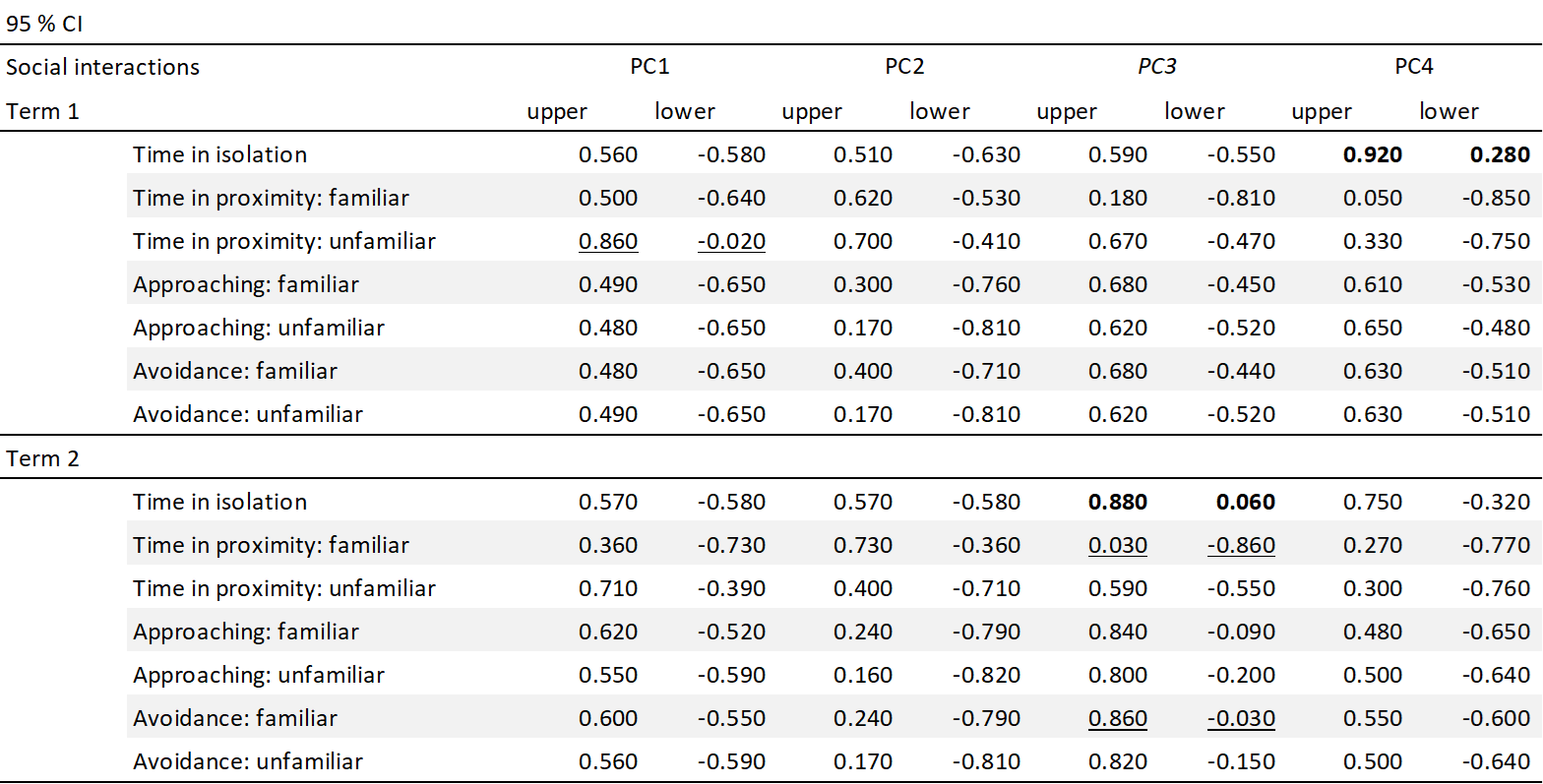

Supplement: S3 Table — Inverted positive/negative values were shown for PC3 for ease of interpretation. (TIF) [file pone.0352127.s002.tif]
